# Refining Long-Term Prediction of Cardiovascular Risk in Diabetes – The VILDIA Score

**DOI:** 10.1038/s41598-017-04935-8

**Published:** 2017-07-05

**Authors:** Georg Goliasch, Günther Silbernagel, Marcus E. Kleber, Tanja B. Grammer, Stefan Pilz, Andreas Tomaschitz, Philipp E. Bartko, Gerald Maurer, Wolfgang Koenig, Alexander Niessner, Winfried März

**Affiliations:** 1 Division of Cardiology, Department of Internal Medicine II, Medical University of Vienna, Vienna, Austria; 2 Department of Internal Medicine, Division of Angiology, Medical University of Graz, Graz, Austria; 3 Medical Clinic V (Nephrology, Hypertensiology, Endocrinology, Diabetology, Rheumatology), Medical Faculty Mannheim, University of Heidelberg, Heidelberg, Germany; 4 Department of Internal Medicine, Division of Endocrinology and Metabolism, Medical University of Graz, Graz, Austria; 5 Bad Gleichenberg Clinic, Bad Gleichenberg, Austria; 6 Department of Internal Medicine, Division of Cardiology, Medical University of Graz, Graz, Austria; 7 Deutsches Herzzentrum München, Teschnische Universität München, Munich, Germany; 8 DZHK (German Centre of Cardiovascular Research), partner site Munich Heart Alliance, Munich, Germany; 9 Department of Internal Medicine, Division of Cardiology, University of Ulm, Ulm, Germany; 10 Clinical Institute of Medical and Chemical Laboratory Diagnostics, Medical University of Graz, Graz, Austria; 11 Synlab Academy, Synlab Services GmbH, Mannheim, Germany

## Abstract

Cardiovascular risk assessment in patients with diabetes relies on traditional risk factors. However, numerous novel biomarkers have been found to be independent predictors of cardiovascular disease, which might significantly improve risk prediction in diabetic patients. We aimed to improve prediction of cardiovascular risk in diabetic patients by investigating 135 evolving biomarkers. Based on selected biomarkers a clinically applicable prediction algorithm for long-term cardiovascular mortality was designed. We prospectively enrolled 864 diabetic patients of the LUdwigshafen RIsk and Cardiovascular health (LURIC) study with a median follow-up of 9.6 years. Independent risk factors were selected using bootstrapping based on a Cox regression analysis. The following seven variables were selected for the final multivariate model: NT-proBNP, age, male sex, renin, diabetes duration, Lp-PLA2 and 25-OH vitamin D3. The risk score based on the aforementioned variables demonstrated an excellent discriminatory power for 10-year cardiovascular survival with a C-statistic of 0.76 (P < 0.001), which was significantly better than the established UKPDS risk engine (C-statistic = 0.64, P < 0.001). Net reclassification confirmed a significant improvement of individual risk prediction by 22% (95% confidence interval: 14–30%) compared to the UKPDS risk engine (P < 0.001). The VILDIA score based on traditional cardiovascular risk factors and reinforced with novel biomarkers outperforms previous risk algorithms.

## Introduction

There is broad evidence that diabetes mellitus is associated with elevated all-cause and cardiovascular mortality^1–3^. However, the increase in risk associated with diabetes mellitus varies considerably^4^. Based on novel biomarkers great efforts have been made to improve risk estimation in people with cardiovascular disease^5, 6^. Nevertheless, an up-to-date risk score specifically addressing people with diabetes is not available. Hence, the most widely used algorithm for people with diabetes mellitus is still the U.K. Prospective Diabetes Study (UKPDS) risk score^7^. This algorithm only includes two biomarkers, namely glycated hemoglobin and the total cholesterol to high-density lipoprotein cholesterol ratio^7^. Since the development of the UKPDS score numerous biomarkers have been found to be independent predictors of cardiovascular disease, for example C-reactive protein, Vitamin-D, and renin^8^.

Hence, the present analyses aimed to improve the prediction of cardiovascular death in diabetic patients using a variety of evolving biomarkers and to show superior prognostic accuracy compared with the UKPDS risk score and compared with single predictors. The initial choice of the biomarkers was based on the multifactorial pathogenesis of cardiovascular disease in patients with diabetes and the routine availability of lab assays to facilitate a rapid clinical translation. The choice of the final biomarker set was based on individual predictive power in the univariate screen. We studied participants of the LUdwigshafen Risk and Cardiovascular health (LURIC) study who had a known personal history of diabetes mellitus or were newly diagnosed with diabetes mellitus^9^. LURIC is a large prospective observational study which was designed to investigate clinical and biochemical cardiovascular risk factors^9^. Potential clinical implications of improved risk stratification are a tailored use of preventive strategies with a particular focus on high-risk patients.

## Results

### Baseline characteristics and univariate survival analysis

We prospectively enrolled 864 diabetic patients of the LURIC study into our study. Forty-three percent of all patients (n = 369) died during a median follow-up of 9.6 years (IQR: 5.5–10.4) corresponding to 6837 overall person-years of follow-up. Sixty-eight percent of deaths in patients were due to cardiovascular causes. Thirty-five percent of patients experienced myocardial infarction before study inclusion. Detailed baseline characteristics of the study population are displayed in Supplemental Table 1.

In the univariate analysis, the strongest adverse risk factors for long-term outcome in diabetic patients were NT-proBNP with a crude hazard ratio (HR) of 2.04 (95% confidence interval [CI] 1.82–2.28; P < 0.001) per 1-SD increase and age (crude HR 1.51; 95% CI 1.35–1.70; P < 0.001; Table 1). 25-OH vitamin D_3_ had a strong inverse effect on mortality with a HR of 0.68 per 1-SD increase (95% CI 0.61–0.75 P < 0.001; Table 1).

**Table 1:** Unadjusted and adjusted effects on all-cause mortality. Cox proportional hazard model of variables selected by bootstrapping. Hazard ratios (HR) refer to a 1-SD increase in continuous variables, a reduction in one category of LVEF and a increase in one category of diabetes duration. HRs are adjusted (adj.) for all variables selected by bootstrapping i.e. for male sex, age, pro-BNP, HbA1c, renin, 25-OH vitamin D_3_, and lipoprotein associated phospholipase A2 (Lp-PLA2). SD – standard deviation.

Variables	SD	Crude HR (95% CI)	P-value	Adj. HR (95% CI)	P-value
Nt-proBNP	2395.24	2.04 (1.82–2.28)	**<0.001**	1.83 (1.62–2.06)	**<0.001**
Age	9.12	1.51 (1.35–1.70)	**<0.001**	1.33 (1.18–1.50)	**<0.001**
Male sex	—	1.48 (1.17–1.87)	**0.001**	1.74 (1.37–2.23)	**<0.001**
Renin	244.84	1.47 (1.34–1.62)	**<0.001**	1.40 (1.27–1.54)	**<0.001**
Diabetes duration	—	1.29 (1.19–1.41)	**<0.001**	1.24 (1.14–1.36)	**<0.001**
Lp-PLA2	131.86	1.20 (1.08–1.33)	**<0.001**	1.26 (1.13–1.40)	**<0.001**
25-OH vitamin D_3_	8.64	0.68 (0.61–0.75)	**<0.001**	0.75 (0.67–0.83)	**<0.001**

### Bootstrapping results and multivariable survival analysis

**Figure 1:**
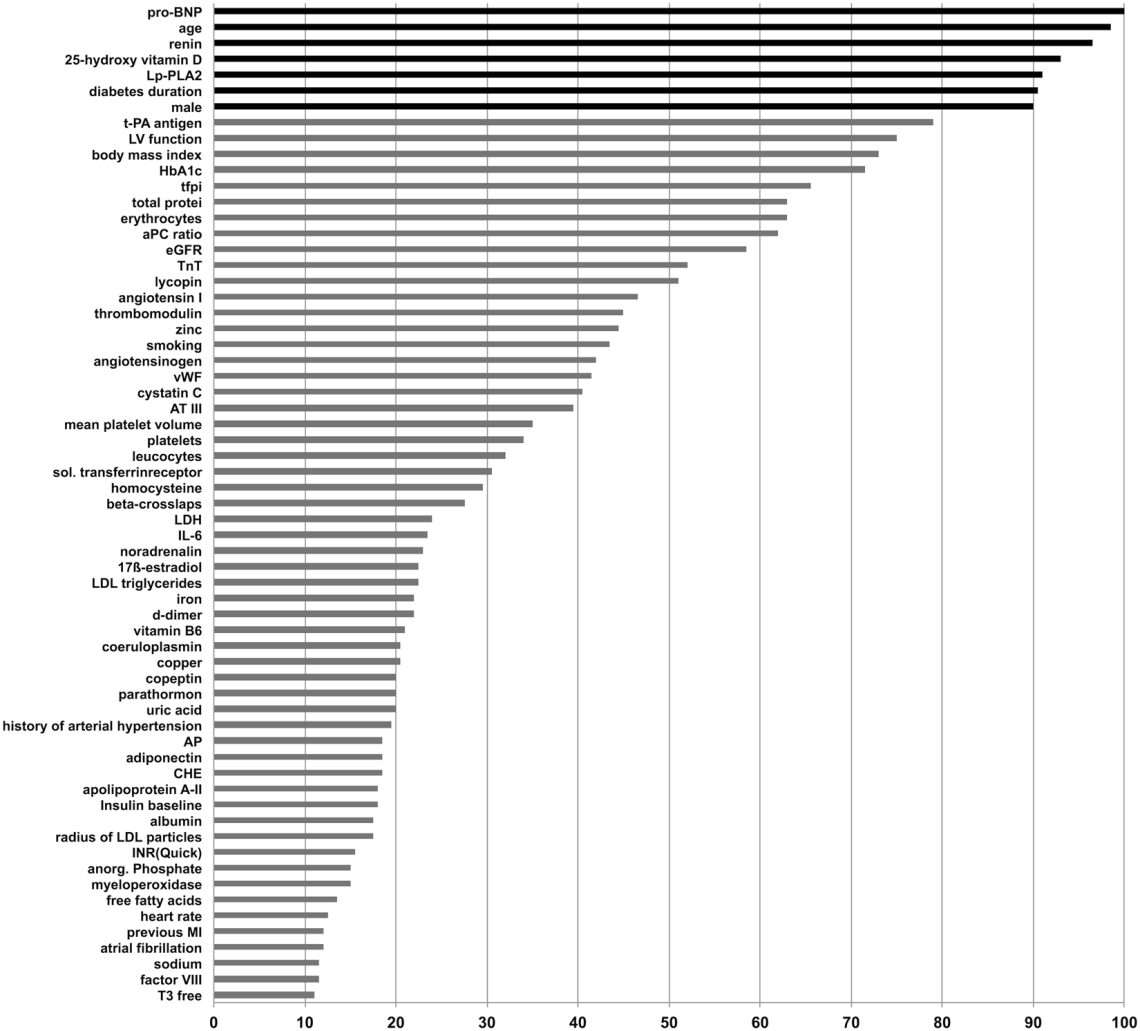
Variable selection by a bootstrap resampling procedure based on Cox regression analysis (cut off level for selection: 80%). To avoid collinearity the following variables were selected for the bootstrap procedure due to their highest univariate predictive value (defined by the hazard ratio for 1-SD change derived from Cox regression) from clusters with close correlations (r > 0.4): Cystatin (NOT selected: BUN, creatinine, ADMA, SDMA, eGFR), free fatty acids, LDL-triglycerides and Lp-PLA2 (NOT selected: total cholesterol, HDL-triglycerides, lipoprotein(a), free glycerol, apolipoprotein A-I, HDL-cholesterol ester, HDL-phospholipid, HDL-cholesterol, cholesterol ester, LDL-cholesterol, apolipoprotein E, LDL-apolipoprotein B, apolipoprotein B, LDL phospholipid, triglycerides, HDL free cholesterol, apolipoprotein C-III, a-Tocopherol, phospholipid, VLDL apolipoprotein B, VLDL-cholesterol ester, VLDL-cholesterol, LDL free cholesterol, VLDL free cholesterol, apolipoprotein A-II), body mass index (NOT selected: waist-to-hip ratio), antithrombin III (NOT selected: retinol), lycopin (NOT selected: alpha-carotin, beta-carotin, lutein, zeaxanthin, all-trans beta-carotin, cis-beta-carotin, beta-cryptoxanthin), noradrenalin (NOT selected: adrenalin), iron (NOT selected: ferritin, transferrin), erythrocytes (NOT selected: hematocrit, hemoglobin, MCV, MCH, MCHC), factor-VII (NOT selected: activated factor-VII), LH (NOT selected: FSH), coeruloplasmin, IL-6 (NOT selected: fibrinogen, serum amyloid A, LBP, hsCRP), INR(Quick) (NOT selected: endogenous thrombin potential (ETP), prothrombin fragment 1 + 2, aPTT), glycosylated hemoglobin (NOT selected: fasting glucose, glucose 1 and 2 h post OGTT, fasting insulin, insulin 1 and 2 h post OGTT, proinsulin 1 and 2 h post OGTT, C-peptide 1 and 2 h post OGTT), cholinesterase (NOT selected: gamma-GT, ALT, AST, bilirubin), osteocalcin, beta-crosslaps, t-PA antigen (NOT selected: t-PA activity, PAI-1 activity, PAI-1 antigen), NT-proBNP (NOT selected: homoarginine), renin (NOT selected: aldosterone), free T3 (NOT selected: free T4, TSH), vitamin-B6 (NOT selected: folic acid, vitamin-B12, vitamin-B1), 25-hydroxy vitamin D (NOT selected: 1–25-dihydroxy vitamin), atrial fibrillation, previous myocardial infarction and duration of diabetes; Variables with a high univariate hazard ratio (NT-proBNP, TnT, cystatin C, eGFR, copeptin) were included in the bootstrapping resampling procedure despite correlation coefficients >0.4. In case of close correlations selection of the respective variables were tested with and without the correlating variable. quick prothrombin time; BUN, blood urea nitrogen; HDL, high-density lipoprotein; BMI, body mass index; LDL, low-density lipoprotein; ALT alanin-aminotransferase; hsCRP, high-sensitivity C-reactive protein.

Variables for the multivariate model were selected using a bootstrapping resampling procedure. NT-proBNP, age, male sex, renin, diabetes duration, Lp-PLA2 and 25-OH vitamin D_3_ were selected as significant mortality predictors for the final multivariate model (Fig. 1). In this multivariable model male sex and NT-pro-BNP were the strongest adverse risk factors and 25-OH vitamin D_3_ remained the strongest inverse risk factor (Table 1).

### Risk score design and comparison with established risk scores

Furthermore, we generated the VIenna Ludwigshafen DIAbetes (VILDIA) risk score, a weighted risk score for long-term survival using the aforementioned variables obtained by the bootstrapping procedure (*see supplemental method 1 for the formula*). The C-statistic of the VILDIA score indicated an excellent discriminatory power for 10-year survival with a value of 0.75 (P < 0.001) for all-cause mortality and for 10-year survival free of cardiovascular death with a value of 0.76 (P < 0.001). The P values for the Grønnesby and Borgan statistics indicated a good calibration for the VILDIA score for all-cause and cardiovascular mortality models (P > 0.20). The discriminatory ability of our score was superior to the previously published UKPDS risk engine^7^ for 10-year survival free of cardiovascular death (C-statistic 0.64; P < 0.001 for comparison). Furthermore, the VILDIA score was significantly superior to the UKPDS risk engine with respect to improved risk classification with a NRI of 21% (95% CI: 12–30%; P < 0.001) for all-cause mortality (Table 2) and of 22% (95% CI: 14–30%; P < 0.001) for cardiovascular mortality (Table 3).

**Table 2:** Reclassification table. Reclassification table comparing the VILDIA score with the UKPDS score for all-cause mortality.

	UKDPS	VILDIA score			
	Risk class	Low	Moderate	High	Total
No event (n = 495)	Low	67% (145)	26% (56)	7% (15)	100% (216)
	Moderate	42% (74)	45% (78)	13% (23)	100% (175)
	High	23% (24)	43% (45)	34% (35)	100% (104)
Event (n = 369)	Low	35% (25)	32% (23)	33% (24)	100% (72)
	Moderate	12% (14)	40% (45)	48% (54)	100% (113)
	High	3% (6)	22% (41)	75% (137)	100% (184)

**Table 3:** Reclassification table. Reclassification table comparing the VILDIA score with the UKPDS score for cardiovascular mortality.

	UKDPS	VILDIA score			
	Risk class	Low	Moderate	High	Total
No event (n = 619)	Low	66% (154)	25% (60)	9% (21)	100% (235)
	Moderate	38% (82)	55% (97)	17% (38)	100% (217)
	High	16% (26)	33% (56)	51% (85)	100% (167)
Event (n = 245)	Low	30% (16)	36% (19)	34% (18)	100% (53)
	Moderate	8% (6)	37% (26)	55% (39)	100% (71)
	High	3% (4)	25% (30)	72% (87)	100% (121)

Data are given as % row (n).

**Figure 2:**
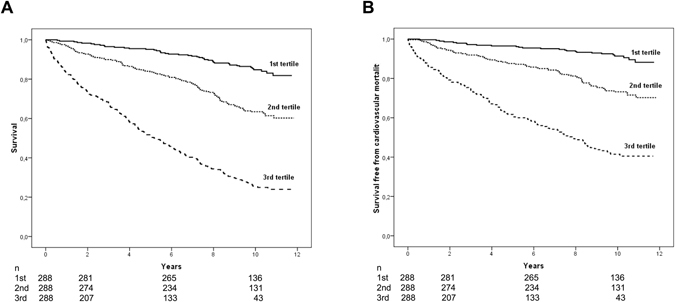
Kaplan-Meier estimates of mortality. Kaplan-Meier estimates for tertiles of VILDIA score for all-cause mortality (**A**) and cardiovascular mortality (**B**) (all P < 0.001 between all tertiles, log rank test).

Kaplan Meier analysis confirmed the high discriminatory value when plotting tertiles of the new risk score for all-cause mortality (Fig. 2A) and cardiovascular mortality (Fig. 2B). The 10-year survival rates in the first, second and third tertile of the new score were 85%, 63%, and 25%, respectively (P < 0.001 between all tertiles). With regard to cardiovascular mortality, a comparable trend for the 10-year survival was observed, with survival rates of 91%, 73%, and 41% in the first, second and third tertile of the new risk score (P ≤ 0.001 between all tertiles).

### Simplified score

**Figure 3:**
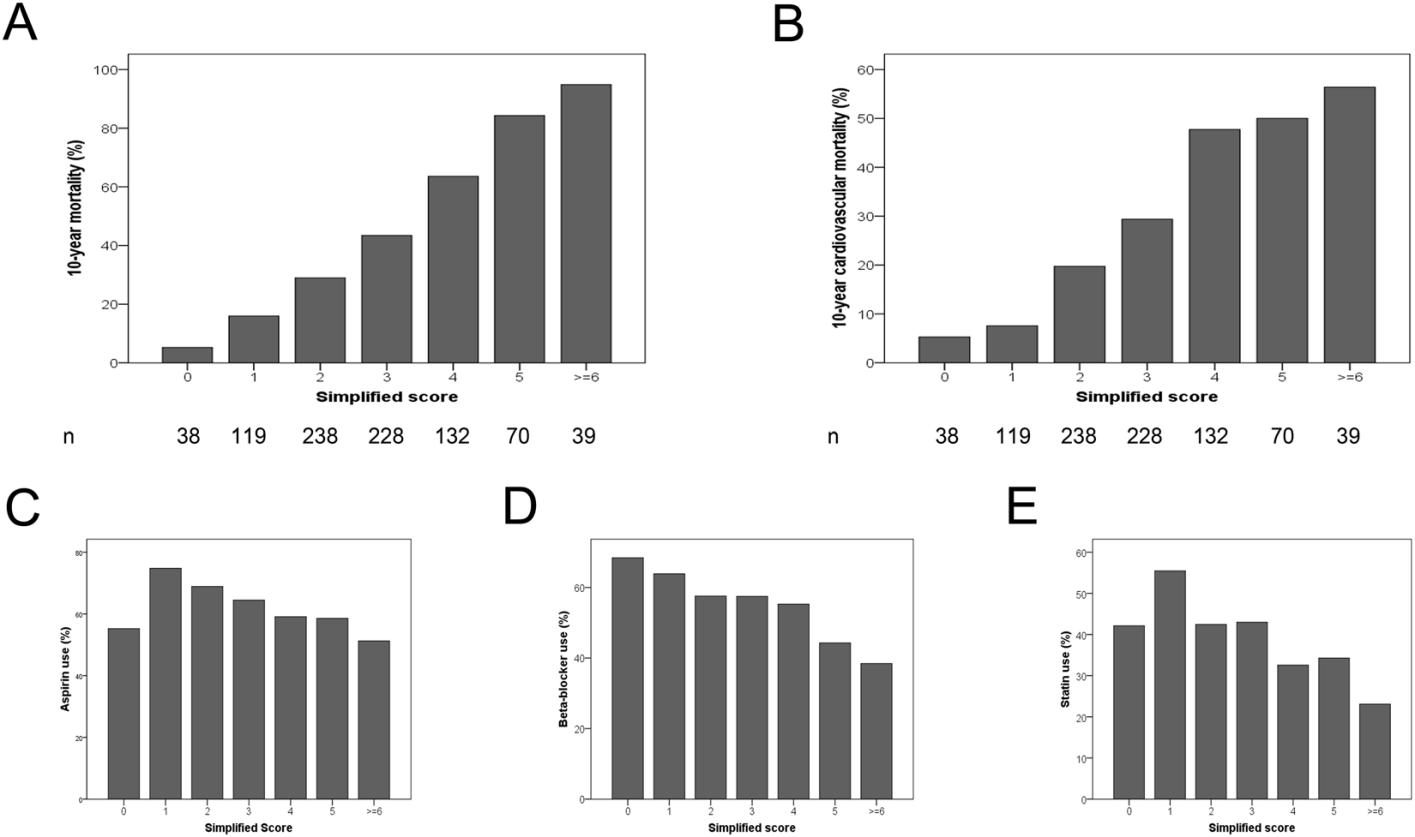
Risk prediction of simplified risk score and association with established cardiovascular medication. The bars show 10-year all-cause (**3A**) and cardiovascular (**3B**) mortality stratified by the simplified biomarker score. We further found an inverse associations between the simplified risk and aspirin use (for trend, p < 0.007, Fig. 3C), beta blocker use (for trend, p < 0.001, Fig. 3D) and statin use (for trend, p < 0.001, Fig. 3E) at discharge. The simplified score was based on the following cut-off values: [male = 1] + [age ≥ 75years (86^th^ percentile) = 1] + [NT-proBNP ≥ 400 ng/L (51^th^ percentile) = 1, ≥2000 ng/L (85^th^ percentile) = 2] + [diabetes duration ≥ 5 years (81^th^ percentile) = 1] + [renin ≥ 50 pg/ml (74^th^ percentile) = 1)] + [25-OH vitamin D_3_ < 10 ng/L (29^th^ percentile) = 1] + [Lp-PLA2 > 450 U/l (43^rd^ percentile) = 1].

Ultimately, we aimed to create a simplified VILDIA score for optimal clinical use. The design of this simplified score was based on the same variables as the weighted risk score. Continuous variables were dichotomized according to optimal cut-off levels, as described in Fig. 3. Two points were assigned to the variable Nt-proBNP with the strongest predictive value in the multivariate model while one point was allocated to the other variables. The simplified VILDIA score still demonstrated a strong discriminatory power with a C-statistic of 0.72 for all-cause mortality and 0.72 for cardiovascular mortality over 10 years. The discriminatory ability of the simplified VILDIA score was still superior to the UKPDS risk engine (P = 0.01). All-cause mortality ranged from 5.3% with zero points to 94.9% with six or more points (Fig. 3A). Cardiovascular mortality ranged from 5.3% to 56.4% (Fig. 3B). With regard to the use of pharmacologic treatment reducing cardiovascular mortality we found inverse associations between the simplified VILDIA score and aspirin use (for trend, p < 0.007, Fig. 3C), beta blocker use (for trend, p < 0.001, Fig. 3D) and statin use (for trend, p < 0.001, Fig. 3E) at discharge.

## Conclusions

We developed the VILDIA score, a multimarker score for patients with diabetes, which provides an excellent discriminatory power for the prediction of 10-year survival with a C-statistic of 0.75. The final set of seven variables was selected using a bootstrapping resampling procedure from a large set of 135 clinical and biochemical markers encompassing various pathophysiologic pathways and reflecting different aspects of the vulnerable patient with diabetes. The clinical relevance of this new multimarker score was further emphasized by a significant improvement of net reclassification by 21%, which reflects the proportion of individuals, who may benefit from an improved risk prediction compared to the regularly used UKPDS risk engine. The new VILDIA score predicted long-term mortality irrespective of the presence of a previous MI.

In general, diabetes is associated with an approximately three times higher risk of cardiovascular events^10^. Accordingly, similar preventive measures such as stricter target values for cholesterol or blood pressure have been proposed^11^. However, an risk increase of diabetic patients equivalent to established coronary artery disease has been recently questioned^12^. Therefore, a refinement of cardiovascular risk estimation in patients with diabetes is warranted to manage the cardiovascular risk of patients with diabetes in an individualized form. So far the UKPDS risk engine has been the best available tool to predict the cardiovascular risk of patients with diabetes. This score included clinical characteristics including age, sex, ethnicity, smoking, HbA1c, systolic blood pressure and cholesterol^7^. However, the new VILDIA score outperformed this score by adding a set of selected biomarkers. As shown by a superior predictive value of the score compared to single risk markers this score may also reflect the multifactorial pathogenesis of cardiovascular events more accurately than a single marker. Robustness of the selection of markers (Fig. 1) and independency of their predictive value as shown in the multivariable model (Table 1) strengthened the overall predictive value of the score. Furthermore, selection from an initial set of more than 100 biomarkers strongly increases the probability of covering the majority of relevant pathogenetic factors. The predictive value of VILDIA was not influenced by previous MI and, even more importantly, the presence of a previous MI did not increase the predictive value of the VILDIA score (data not shown). Furthermore, as expected, the presence or absence of angiographic coronary artery disease or semi-quantitative measures of the atherosclerotic burden to the coronary arteries did not substantially contribute to risk stratification. This is due to the fact that angiography visualizes the vessel lumen only and does not provide information on the vulnerability of plaques nor on their actual size^13, 14^.

A non-invasive alternative to coronary angiography would be computed tomography of the coronary arteries to visualize and quantify coronary calcium. In a systematic review of current guidelines the authors found that 10 of 14 guidelines considered the CT calcium score as a test for improvement of coronary risk assessment, however only 4 guidelines concluded that there was sufficient evidence for the use of coronary calcium scoring and only 1 guideline recommended its use solely in an intermediate CAD risk population^15^. More importantly for low CAD risk persons and persons already known to be at high CAD risk, guidelines were unanimous in not advocating CT calcium scoring^15^. Yet, it remains to be determined whether coronary calcium scoring would indeed yield superior prognostic information above and beyond the VILDIA algorithm. Finally, an alternative strategy could be to perform cheaper biomarker tests in advance of calcium scoring in a step-wise fashion. It is however, unlikely, that calcium scoring will considerably improve prediction beyond the biomarkers.

Other risk scores to predict CAD risk in type 2 diabetes have been described but to the best of our knowledge the UKPDS risk score is the most powerful tool to assess CAD risk and that is why we focused on comparison to the UKPDS risk engine. The AD-ON score from the ADVANCE trial showed excellent discrimination for major renal events albeit only moderate discrimination for CHD (c-statistic = 0.67) compared to most risk scores^16^. However, the AD-ON score showed good calibration among different regions. This is specifically important as a risk score might not be generally applicable to all regions. For instance Yang and coworker showed that the UKPDS risk engine overestimated CHD risk among Chinese patients with type 2 diabetes^17^. Stable calibration among different geographical regions for the VILDIA score remains to be demonstrated. The look AHEAD research group showed the predictive value of a genetic risk score in obese patients with type 2 diabetes^18^.

The LURIC study participants represent a cohort at intermediate to high cardiovascular risk. At a first glance, our conclusions may therefore not be generalizable to a random population sample. However, global cardiovascular risk in our patients comes very close to that of patients with diabetes mellitus recruited in other settings. For instance, in a population-based study^3^ the proportion of diabetes patients with a history of myocardial infarction was similar to that in our cohort and, more importantly, the overall annual rate of death from cardiovascular causes in diabetes mellitus patients was approximately 2.9 percent while in LURIC it was 2.8 percent. In other population based-studies, very similar incidence rates of cardiovascular deaths have been seen^19–21^. It thus appears that our study population is indeed representative of patients with diabetes mellitus in general, and we speculate that the relatively high baseline cardiovascular risk of the study participants is sufficiently explained by the presence of diabetes mellitus rather than by other reasons such as referral bias. Finally, the comparatively high risk may represent an important strength of the study, because precisely targeted treatment is most likely to yield benefit in these patients and previous risk assessment in the high risk population has been exceptionally inconsistent^22^.

All (bio)markers included in the VILDIA score have been shown to be associated with cardiovascular risk. Duration of diabetes is associated with increased risk of major coronary heart disease events^23^ and has been repeatedly identified as a potent predictor of all-cause and cardiovascular mortality^24–26^. ProBNP and its N-terminal fragment NT-proBNP are one of the strongest and most robust predictors of mortality in the cardiovascular field, particularly in patients with heart failure^27^ and acute coronary syndrome^28, 29^. Natriuretic peptides may also be important predictors of cardiovascular and all-cause mortality in patients with diabetes^30–32^ {Hillis, 2014 #38}. In our diabetes cohort, Nt-proBNP has been the most robust parameter in the bootstrapping resampling procedure, even more robust than left ventricular function measured by echocardiography (Fig. 1). Renin, part of the renin-angiotensin-aldosterone system (RAAS), is not only involved in development of arterial hypertension^33^ but also in the progression of cardiovascular diseases and is associated with cardiovascular events^34, 35^. Conflicting data derived from the Framingham Heart Study where renin was associated with mortality but not with incidence of cardiovascular disease^36, 37^. Furthermore, direct inhibition of renin in addition to standard RAAS inhibition in patients with diabetes has not shown to be beneficial with regard to a composite endpoint including cardiovascular and renal events^38^. 1,25-OH vitamin D_3_, the active form of vitamin D, requires sunlight as well as conversion in the liver and the kidney. 25-OH vitamin D_3_, the major circulating form, may be preferred as biomarker. Apart from its role in skeletal pathologies vitamin D deficiency also affects extraskeletal diseases including cardiovascular diseases. Particularly, in patients with metabolic syndrome or diabetes, low levels of vitamin D have been associated with increased cardiovascular mortality^39, 40^. Finally, activity of Lp-PLA2, also described as platelet-activating factor acetylhydrolase, is closely correlated with cardiovascular risk factors suggesting an important role in atherogenesis^41^. Experimental data indicated that inhibition of Lp-PLA2 may prevent progression of atherosclerotic plaques^42^. Furthermore, Lp-PLA2 is associated with cardiovascular mortality^43^ and predicts cardiovascular outcome in diabetic patients^44^.

While scores are superior in risk prediction compared to single risk factors they are underutilized in clinical practice^6, 45^. An easy-to-use form such as the simplified VILDIA score using categorized variables based on optimized cut-offs is therefore of utmost importance for daily clinical use. The simplified VILDIA score shows a comparable predictive accuracy and is able to fulfill the most important aim of ranking patients^46^ into low 10-year fatal cardiovascular risk (<10%) with zero or one point, intermediate risk (20–30%) with two to three points and high risk (about 50% or more) with four or more points. Our results strongly support that patients with diabetes mellitus are very heterogeneous with regard to their cardiovascular risk. In accordance, it seems obsolete to regard diabetes mellitus simply as a cardiovascular disease risk equivalent. The VILDIA score may provide a novel tool for clinicians, to more easily estimate the risk of patients with diabetes mellitus on the basis of a few clinical and laboratory parameters. The refinement of cardiovascular risk estimation for the individual patients as evidenced by an improved NRI may lead to a more accurate treatment of the individual patient. A negative correlation between the VILDIA score and aspirin, statin and beta-blocker use suggests that there may be potential to optimize the therapy. E.g. less than 30% of patients with a simplified VILDIA score of ≥6 received statins. However, this high-risk group has a 10-year cardiovascular mortality of more than 50%. While there is an ongoing discussion about an increased incidence of diabetes in patients on statins they certainly reduce cardiovascular events in patients with known diabetes^47^. Likewise, aspirin and beta-blockers, both reducing cardiovascular risk, have been prescribed with the lowest frequency in patients with a high VILDIA score suggesting a benefit of an individualized risk management based on the VILDIA score. Nevertheless, little consensus exists concerning the question whether diabetic patients without overt cardiovascular disease should receive antiplatelet therapy^48^. Another open question is whether diabetic patients will benefit from screening for silent coronary artery disease^4^. Trying to answer these questions it seems essential to find those patients, who will most likely profit from expensive screening and intervention strategies. The novel VILDIA-score appears to provide this information at affordable costs.

The limited number of included patients and the lack of external validation is a relevant limitation of this study. However, assessment of more than 100 biomarkers in a larger or second cohort is hardly accomplishable. Replication of the score using selected variables only will be required in an external cohort before clinical use as varying population characteristics may lead to different risk estimates. Additionally, medication at time of study enrollment may have to some extent influenced the recorded biomarker levels. Further, a long-term follow-up implicates that patients have been selected about 10 years ago. Therefore, measures of secondary prevention may be used in a different way nowadays. Another hurdle to the clinical application may be significant costs due to measurement of biomarkers. However, the set has been limited to the most important markers and resulted in a superior predictive value. Cost savings following the implementation of the VILDIA score for efficient prevention of cardiovascular events are likely. However, cost-effectiveness analysis will be needed before implementation. While we anticipate that implementation of such a score would require further considerations of risk and benefits, we believe that a comprehensive cost-benefit simulation would go far beyond the scope of this manuscript. Furthermore, there were notable differences in age range among the present cohort and the UKPDS cohort. Partially this can be attributed to changes in the general population characteristic. The UKPDS however is -up to now- the most broadly used tool for risk stratification and the scores performed equally well within age groups in the present cohort. Importantly, comparison of the predictive value of the scores is also limited as the UKPDS risk engine was initially developed as a predictive model for CAD. Strengths of this study are the comprehensive set of biomarkers and a long-term follow-up of 10 years. Of course the current data are observational in nature and only a randomized trial would have the potential to demonstrate whether risk algorithm guided management of patients with diabetes mellitus improves long term prognosis above and beyond usual care.

In conclusion, the VILDIA score has confirmed the hypothesis that a multi-biomarker score may improve risk estimation in patients with diabetes. Assessment of a set of independent biomarkers may reflect the multi-factorial pathogenesis of cardiovascular events better than single risk factors and scores based on routine variables. A more individualized treatment based on the VILDIA score will provide the potential benefit of improving the therapy of high-risk patients as well as avoiding overtreatment in low-risk patients.

## Research Design and Methods

### Study population

The Ludwigshafen Risk and Cardiovascular Health (LURIC) study comprises 3,316 Caucasian patients referred to the Cardiac Center Ludwigshafen (Germany) for coronary angiography between 1997 and 2000. The detailed study protocol has been previously published^9^. The study protocol complies with the Declaration of Helsinki and was approved by the Ethics Committee of the “Ärztekammer Rheinland-Pfalz”. Written informed consent was collected before study enrollment in all patients. For the present analysis, we chose 864 patients with diabetes. Patients with an acute coronary syndrome at hospital admission were excluded from our analysis.

### Clinical definitions

Diagnosis of diabetes was based on the revised American Diabetes Association criteria^49^. Subjects with increased fasting (≥126 mg/dl) and/or post-challenge (2 h after the 75 g glucose load ≥200 mg/dl) glucose or with an HbA1c > 6.5% were diagnosed with diabetes^50^. People with a self-reported or documented history of diabetes or treatment with oral antidiabetics or insulin were considered diabetic (9). Diabetes duration was categorized into newly diagnosed diabetes, duration ≤5 years, duration >5 to ≤10 years, duration >10 years as previously published^26^. Clinical risk factors such as hypertension, current smoking, lipid disorders and previous myocardial infarction (MI) were diagnosed according to the respective guidelines as previously described^9^. Venous blood samples were drawn under standardized conditions after an overnight fasting period. NT-proBNP was measured by electrochemiluminescence on an Elecsys 2010 (Roche Diagnostics). Plasma renin concentration was determined by an immunoradiometric assay (Active Renin, Nichols Institute Diagnostics, San Juan, Capistrano, CA, USA). Lipoprotein associated phospholipase A2 (Lp-PLA2) activity was measured by use of the Azwell Auto PAF-AH reagent set (Azwell). Serum levels of 25-OH vitamin D_3_ were measured using a radioimmunoassay (DiaSorin SA, Antony, France). Intra and inter-assay coefficients of variation were <10% for these assays.

### Study endpoints and follow-up

The primary study endpoints were all-cause mortality and cardiovascular mortality. Information on patient outcome was obtained by screening local registries. No patient was lost to follow-up. Death certificates were obtained in 97% of deceased participants and used to classify them into those who died of cardiovascular versus non-cardiovascular causes as previously described^9^. This classification was performed independently by two experienced clinicians who were blinded to the study participants, except for information that was required to classify the cause of death.

### Statistical analysis

Discrete data was presented as counts and percentages, continuous data were presented as median and interquartile range. Cox proportional hazard regression analysis was used to evaluate the effect of the investigated variables on survival. Continuous variables were log-transformed before entering analysis to account for not normally distributed variables. For standardization purposes we decided to analyze all variables uniformly. A bootstrap resampling procedure was used to identify best-fitting predictors of all-cause mortality for the final multivariate Cox regression model. Before inclusion of variables into the bootstrapping procedure collinearity between continuous variables was assessed using Pearson correlation coefficients (data not shown). In case of close correlation only the strongest univariate predictor of a cluster (defined by the highest univariate hazard ratio for an increase of 1 standard deviation (SD)) entered the selection procedure. Samples with a size of 100% of the original cohort were chosen for repeats. 100 repeats with backward selection and 100 repeats with forward selection (with a P < 0.1 for selection) were executed stepwise. Variables selected in more than 80% of all repeats were included in the final multivariable model. Harrell’s C-statistic was used to estimate the discriminatory power of the designed risk score. Moreover, the calibration of the score was assessed using the Grønnesby and Borgan statistics, comparing the observed and model-based estimated expected number of events within four risk groups. For further analysis the cohort was stratified by tertiles of the biomarker risk score. An improvement of individual risk prediction was examined by the net reclassification improvement (NRI). Kaplan-Meier analysis (log-rank test) was applied to verify the time-dependent discriminative power of risk scores. Cut-off values for a simplified score using categorized variables were estimated using chi-square values derived from Martingale residuals of the Cox model for all possible cut-off values. For optimized clinical use these calculated cut-off values were rounded and extreme values were avoided. Chi square test for linear to linear association was used to assess the trend between medication and simplified risk score categories. Two-sided P-values < 0.05 were used to indicate statistical significance. The STATA software package (Stata Statistical Software: Release 11, StataCorp LP, USA) and SPSS 17.0 (IBM SPSS, USA) were used for all analysis.

## Electronic supplementary material

Supplementary Information
