# Supplementary material for: Refining Long-Term Prediction of Cardiovascular Risk in Diabetes – The VILDIA Score
Source: Sci Rep. 2017 Jul 5;7:4700. doi: 10.1038/s41598-017-04935-8 (PMC5498499; doi:10.1038/s41598-017-04935-8)
Supplement: Supplementary file 1 — Supplementary Information [file 41598_2017_4935_MOESM1_ESM.doc]

**Supplementals: Refining Long-Term Prediction of Cardiovascular Risk in Diabetes – The VILDIA Score**

Georg Goliasch, MD, PhD^1^; Günther Silbernagel, MD^2^; Marcus E. Kleber, PhD^3^; Tanja B. Grammer, MD^3^; Stefan Pilz, MD^4^; Andreas Tomaschitz, MD^5,6^; Philipp E. Bartko^1^; Gerald Maurer, MD^1^; Wolfgang Koenig, MD^7,8,9^; Alexander Niessner, MD, MSc^1^; Winfried März, MD^3,10,11^;

Affiliations: 1 Division of Cardiology, Department of Internal Medicine II, Medical University of Vienna, Austria 2 Department of Internal Medicine, Division of Angiology, Medical University of Graz, Graz, Austria and 3 Medical Clinic V (Nephrology, Hypertensiology, Endocrinology, Diabetology, Rheumatology), Medical Faculty Mannheim, University of Heidelberg, Heidelberg, Germany, and 4 Department of Internal Medicine, Division of Endocrinology and Metabolism, Medical University of Graz, Graz, Austria, and 5 Bad Gleichenberg Clinic, Bad Gleichenberg, Austria, and 6 Department of Internal Medicine, Division of Cardiology, Medical University of Graz, Graz, Austria, and 7 Deutsches Herzzentrum München, Teschnische Universität München, Munich, Germany and 8 DZHK (German Centre of Cardiovascular Research), partner site Munich Heart Alliance, Munich, Germany, and 9 Department of Internal Medicine, Division of Cardiology, University of Ulm, Ulm, Germany and 10 Clinical Institute of Medical and Chemical Laboratory Diagnostics, Medical University of Graz, Graz, Austria, and 11 Synlab Academy, Synlab Services GmbH, Mannheim, Germany

**Supplemental table 1: Baseline characteristics and biomarkers** - Continuous variables are given as median (interquartile range [IQR]). Left ventricular ejection fraction (LVEF); ECG abnormalities are defined as ST-segment deviation and/or T wave inversion; bpm beats per minute

|  | LURIC study population (n=864) |
| --- | --- |
| Age, years | 66 (59- 72) |
| Male gender, n (%) | 595 (69%) |
| BMI, kg/m^2^ | 27.8 (25.3- 30.6) |
| Systolic blood pressure, mmHg | 146 (129- 162) |
| Heart rate, bpm | 70 (63- 78) |
| LVEF |  |
| ≥55%, n (%) | 496 (62%) |
| 45-54%, n (%) | 125 (15%) |
| 30-44%, n (%) | 125 (15%) |
| <30%, n (%) | 63 (8%) |
| Medication |  |
| Oral anti-hyperglycemic agents, n (%) | 168 (19%) |
| Insulin treatment, n (%) | 113 (13%) |
| Beta blocker, n (%) | 489 (57%) |
| ACE inhibitor/Angiotensin II  receptor blockers, n (%) | 520 (60%) |
| Statins, n (%) | 357 (41%) |
| Hypertension, n (%) | 564 (65%) |
| Current smokers, n (%) | 167 (19%) |
| Previous myocardial infarction, n (%) | 304 (35%) |
| Previous stroke, n (%) | 107 (12%) |
| Diabetes mellitus type 1, n (%) | 7 (1%) |
| Diabetes mellitus type 2, n (%) | 857 (99%) |
| Hba1c, mg/dl | 6.8 (6.5- 7.8) |
| Glucose, mg/dl | 109 (92- 137) |
| Basal insulin, µg/ml | 11 (7- 19) |
| Diabetes duration |  |
| Duration > 10 years, n (%) | 108 (13%) |
| Duration > 5 to ≤ 10 years, n (%) | 57 (7%) |
| Hypercholesterolemia, n (%) | 498 (58%) |
| Total cholesterol, mg/dl | 190 (164- 215) |
| VLDL cholesterol, mg/dl | 33 (22- 51) |
| LDL cholesterol, mg/dl | 112 (91- 136) |
| HDL cholesterol, mg/dl | 36 (31- 43) |
| Triglycerides, mg/dl | 156 (115- 218) |
| VLDL triglycerides, mg/dl | 109 (71- 167) |
| LDL triglycerides, mg/dl | 30 (24- 38) |
| HDL triglycerides, mg/dl | 15 (11- 20) |
| Lp (a), mg/dl | 10 (7- 33) |
| ApoA-I, mg/dl | 126 (112- 143) |
| ApoA-II, mg/dl | 40 (34- 46) |
| Average LDL particle radius, nm | 8.26 (8.12- 8.40) |
| Free fatty acids, mmol/L | 0.68 (0.50- 0.98) |
| Zinc, µmol/L | 86 (77- 96) |
| Iron, µg/dl | 89 (68- 115) |
| Sodium, mmol/L | 141 (139- 143) |
| Copper, µg/dl | 107 (92- 124) |
| Coeruloplasmin, mg/dl | 30 (26- 34) |
| Soluble transferrin receptor, mg/L | 1.28 (1.06- 1.58) |
| Phosphate, mg/dl | 3.5 (3.2- 3.9) |
| Beta-crosslaps, ng/ml | 0.28 (0.18-0.42) |
| Creatinine, mg/dl | 0.9 (0.8- 1.1) |
| Cystatin C, mg/L | 1.0 (0.8- 1.2) |
| Estimated GFR, ml/min/1.73 m^2^ | 84 (67- 104) |
| Renin, U/L | 22 .5 (11- 53) |
| Angiotensin I | 1499 (1116- 1897) |
| Angiotensinogen, nmol/L | 1158 (862- 1464) |
| Uric acid, mg/dl | 5.1 (4.2- 6.3) |
| Protein, g/l | 6.9 (6.6- 7.2) |
| Albumin, g/l | 44 (41- 48) |
| Vitamin B6, µg/L | 8.2 (5.2- 13) |
| 25OH-vitamin D_3_, µg/L | 14.2 (9.3- 20.6) |
| Cholinesterase, kU/L | 5.80 (4.83- 6.65) |
| Alkaline phosphatase, U/L | 118 (97- 144) |
| Noradrenalin, ng/L | 329 (232- 471) |
| 17β estradiol, ng/L | 37 (27- 48) |
| Lactate dehydrogenase, U/L | 169 (148- 192) |
| Parathyroid hormone, pg/ml | 31 (23- 42) |
| Lycopin, µmol/L | 0.23 (0.14- 0.36) |
| Homocysteine, µmol/L | 12.6 (10.2- 15.7) |
| Lp-PLA2, U/L | 473 (392- 563) |
| IL-6, ng/L | 3.4 (2.1- 6.1) |
| C-reactive protein, mg/dl | 0.43 (0.15- 1.01) |
| aPC ratio | 4.5 (4- 4.9) |
| APTT, sec | 33 (30- 36) |
| Antithrombin III, % | 97 (88- 106) |
| INR quick | 1.05 (1.00- 1.11) |
| Factor VII, U/dl | 122 (104- 139) |
| Thrombomodulin, µg/L | 47 (36- 61) |
| vWF, U/dl | 170 (130- 210) |
| D-dimer, mg/L | 0.36 (0.22- 0.63) |
| Tissue factor pathway inhibitor, µg/L | 1.22 (1.00- 1.43) |
| PAI-1 antigen, µg/L | 30 (18.5- 44.6) |
| tPA antigen, µg/L | 13 (10.2- 16.1) |
| Platelets, 1000/µl | 217 (184- 263) |
| Mean platelet volume, fl | 9.0 (8.4- 9.7) |
| Erythrocytes, 10^12^/L | 4.62 (4.3- 4.9) |
| Leukocytes, 1000/µl | 6.9 (5.8- 8.4) |
| fT3, pmol/L | 4.8 (4.3- 5.3) |
| TnT, pg/ml | 12.0 (6.5- 24.0) |
| Adiponectin, µg/ml | 8.4 (5.6- 12.8) |
| NT-proBNP, ng/L | 383 (139- 1119) |

**General:**

Weighted risk score = [V1 – mean of V1] * beta coefficient of V1 + [V2 – mean of V2] * beta coefficient of V2 + [V3 – mean of V3] * beta coefficient of V3 + ……..+ [Vn – mean of Vn] * beta coefficient of Vn.

**VILDIA Score:**

= 0.56 * male sex + 0.22 * diabetes duration + 1.95 * (age – 4.17) + 0.41 * (pro-BNP- 5.97) + 0.26 * (renin- 3.28) + 0.85 * (Lp-PLA2– 6.15) – 0.52 * (25-OH vitamin D_3_ – 2.60)

Beta coefficients were derived from a multivariable Cox regression model including all variables of the respective model. Continuous variables were log-transformed before inclusion in the score; n, number of variables of the respective risk score; V, variable
